# A-Maze-Ox: a novel gas-exchange-area-adjustable oxygenator for extremely preterm infants—design and proof of concept

**DOI:** 10.1038/s41390-025-04740-4

**Published:** 2026-01-17

**Authors:** Franziska Schubert, Jan Heyer, Maximilian Lunemann, Jutta Arens, Ulrich Steinseifer, Sebastian Victor Jansen, Mark Schoberer

**Affiliations:** 1 Department of Cardiovascular Engineering, Institute of Applied Medical Engineering, Medical Faculty, RWTH Aachen University, Aachen, Germany; 2 Engineering Organ Support Technologies, Faculty of Engineering Technology, Department of Biomechanical Engineering, University Twente, Enschede, Overijssel Netherlands; 3 Pediatric Clinic, Neonatology Section, Medical Faculty, RWTH Aachen University and University Hospital, Aachen, Germany

## Abstract

**Background:**

Each year, over half a million children worldwide are born extremely prematurely (EPI), often requiring potentially harmful ventilatory support. An artificial placenta with gas exchange provided by an oxygenator offers an alternative. However, current oxygenators are not designed for long-term applications in patients experiencing growth. Thus, a new type of “growing” oxygenator is needed.

**Methods:**

We developed the A-Maze-Ox, a novel maze-inspired membrane oxygenator featuring two concentric compartments that can be opened sequentially to address patient growth. Each compartment has a priming volume of 5 mL and a gas exchange area of 0.065 m². The prototype was tested for feasibility, gas transfer performance and pressure loss according to ISO 7199.

**Results:**

Adding the second compartment increased O_2_ and CO_2_ transfer performance by 57.06% and 35.59%, respectively. While CO_2_ transfer was mostly sufficient, O_2_ transfer remained below the target. The targeted maximum pressure drop of 20 mmHg was exceeded at 90 mL/min.

**Conclusions:**

The A-Maze-Ox demonstrates the ability to adapt to increasing demands with good results in CO_2_ elimination but requires improvement in terms of O_2_ transfer and pressure drop. However, it shows the potential for a growing oxygenator in future artificial placenta applications.

**Impact:**

- Current oxygenators cannot adapt to the dynamic patient growth of extremely premature infants.
- A-Maze-Ox—a novel membrane oxygenator with two compartments can adapt to growth.
- Each compartment has a volume of 5 mL and a gas exchange area of 0.065 m².
- Oxygen transfer increased by 57.06% and carbon dioxide transfer by 35.59% when adding the second compartment.
- A-Maze-Ox could improve health of extremely premature infants in the future.

## Introduction

Preterm birth, defined as birth before 37 weeks’ gestational age (GA), affects approximately 15 million newborns worldwide.^1^ The infants who survive this premature transition to life ex utero often suffer from short- and long-term medical conditions, with increasing risk the earlier the birth occurs.^1–3^ These prematurity-related pathologies include among others early- and late-onset sepsis, intrventricular hemorrhage, necrotizing enterocolitis, seizures, and retinopathy of prematurity.^1,3^ Children born before 28 weeks’ GA are called extremely preterm infants (EPI).^4^ Approximately 4% of all preterm births belong to this category, representing a total of around 600,000 EPI worldwide in 2014.^1^ The degree of organ immaturity in this patient group regularly demands the provision of supportive care on specialized neonatal intensive care units. According to the NICHD Neonatal Research Network survey from 2003 to 2007,^3^ about 62% of all EPI require intubation and mechanical ventilation due to underdeveloped lungs. While being vitally important, this treatment induces lung injury and promotes inflammation leading to disorders of lung development, most importantly bronchopulmonary dysplasia.^5^ Advances in perinatal and neonatal intensive care have improved clinical outcomes over the past decades; however, preterm birth continues to be the leading cause of death among children under the age of 5 years worldwide, accounting for 18% of all deaths in this age group, totaling 1 million deaths every year. Prematurity is responsible for up to 35% of all deaths among newborns (aged less than 28 days).^6^ Consequently, its contribution to neonatal mortality and morbidity remains an important global health issue.^1,7,8^

Over decades, several groups have proposed the concept of an artificial placenta (AP) with extracorporeal life support (ECLS); see Blauvelt et al.^7^ Schoberer et al.^9^, and van Galen et al.^10^ for an overview. In an AP, an extracorporeal oxygenator connected to the umbilical vessels (drainage can also happen via the jugular vein) ensures blood gas exchange such that (mechanical) ventilation is avoided or assisted. The AP can be applied either before or after the onset of spontaneous breathing. In concepts that aim to start treatment before the initiation of breathing, the lungs remain filled with fluid. This can be achieved in two ways: by submerging the infant entirely in artificial amniotic fluid within an “artificial womb” (AW), or by sealing the liquid-filled lungs against the atmosphere using an endotracheal tube (see, for example, ref.^11^), in which case the infant can be placed in a conventional incubator. Both approaches enable the lungs to mature without being involved in gas exchange, thus preserving the fetal situation.

While ECLS is a well-established procedure in full-term neonates, it has not been widely used in premature infants yet and guidelines from the Extracorporeal Life Support Organization even list a GA <34 weeks as a relative contraindication to ECLS.^7,12^ The small body-size of EPI and their immaturity pose challenges to the development of extracorporeal devices integrated into the blood circulation, like the oxygenator. In particular, the increased risk of circulatory clotting, the high risk of cerebral hemorrhage (germinal matrix hemorrhage/intraventricular hemorrhage GMH/IVH)^13^ and the limited ability to compensate for a reduction in blood volume must be considered.^7^

Reduction of the extracorporeal filling volume is key^7,14^ to reduce foreign-surface contact and blood loss. In this regard, Arens et al.^15^ and Omecinski et al.^16^ have developed dedicated small oxygenators: the NeonatOx and the Preemie-Ox with priming volumes of 12 mL and 15 mL, respectively. The customized oxygenator used in the AP system by Miura et al.^17,18^ has a priming volume of 32 mL. The Exp-Ox, recently developed by Kosaka et al.^19^ and integrated into the EXTEND System, features a priming volume of 27 mL. While these are the only published custom-built hollow-fiber membrane (microfluidic oxygenators are not considered here due to their different design - for an overview of corresponding devices in AP technology, please refer to Blauvelt et al.^7^) oxygenators for the use in AP or AW applications, other groups have used or modified already existing devices. For example, Partridge et al.^20^ used the Quadrox-iD Pediatric and a modified Quadrox-i Neonatal with 81 mL and 38 mL priming volume, respectively, in their tests on extremely premature lambs. However, all these concepts are still too large for EPI and none of them take into account that the infant grows rapidly and accordingly has an increasing blood flow and an increasing demand for gas exchange area. The exceptional growth dynamics of this group of patients become even more relevant the longer the envisioned treatment durations are: the oxygenator presented herein considers a four-week treatment period (24- to 28-week of GA), during which weight doubles from 670 g to 1210 g^21^ and blood flow increases from 66.6 mL/min to 110.4 mL/min.^22^ Given the current state of technology, the use of a custom-made oxygenator with minimal foreign-surface area adapted to the needs of a 24-week EPI would require a device change during the course of treatment to accommodate the necessary increase in gas exchange area. This interruption of circulation, however, would result in blood loss, risk of microbiological contamination,^23^ and hemodilution. Connecting an additional oxygenator to the extracorporeal circuit, instead of changing to a device with a larger gas exchange area, is a potential solution to this problem. The most straightforward approach would be to connect an oxygenator that was previously attached in parallel to the primary oxygenator, primed and then disconnected using clamps. However, the additional priming volume required for the second oxygenator and its tubing leads to a comparable degree of hemodilution to that encountered when changing devices. An additional oxygenator compartment within a single device can avoid at least the hemodilution caused by the tubing. From a historical perspective, the idea of a multi-compartment oxygenator was first realized in the VPCML device.^24^ However, the intended use of this two-compartment plate-membrane oxygenator was to address the needs of different patient groups—from children to small adults—within a single system. The priming volume was therefore considerably higher at 425 mL,^25^ and the choice of which compartment to use was typically made prior to application^24^ rather than during ongoing treatment, as is envisioned for our device. Until now, the multi-compartment design has not been adopted in modern hollow-fiber membrane oxygenators, making this consideration a novel approach. In this context, our group has recently proposed a new manufacturing process^26^ that enables the fabrication of an oxygenator based on stacked fiber mats with individual compartments. The dual-compartment design allows to increase the gas exchange area within the device during operation. With very small priming volumes of only 4.72 mL in the inner and 4.5 mL in the outer compartment (higher volumes when including inlet and outlet geometry) and a low pressure drop, initial gas transfer tests showed very promising results regarding oxygen transfer but still scope for improvement in terms of CO_2_-removal. While we revise the oxygenator presented in,^26^ we herein introduce the A-Maze-Ox, an alternative, maze-inspired design for a dual-compartment oxygenator that also allows adjustment of the gas exchange area. The novel design is now based on cylindrical, wound fiber bundles typically providing better CO_2_ elimination rates due to counter current gas and blood flow directions.^27^

## Materials and methods

### Physiological requirements

By combining an extensive literature review with the medical expertise of our Neonatologists, we defined the basic physiological requirements for the design of the oxygenator. The derived values are listed in Table 1 and described below.

**Table 1:** Blood parameters for a 24^th^ and a 28^th^ week of GA infant and derived requirements for a designated oxygenator (bold); some values apply for the entire range of GAs and are therefore only given once.

Gestational age (GA)	weeks	24	28
Fetoplacental blood volume^a^^29^	mL	55	96
Fetal blood volume remaining in the placenta^b28^	%	~50	~40
Max. extracorporeal circuit volume	mL	20	
Mean UV flow^a^^22^	mL/min	66.6	110.4
Derived flow range	mL/min	50	150
pO_2_ in UA^35^	mmHg	19	
pO_2_ in UV^35^	mmHg	31	
sO_2_ in UA^35^	%	38	
sO_2_ in UV^35^	%	72	
Max. sO_2_ in UV^36^	%	82	
pCO_2_ in UA^35^	mmHg	51	
pCO_2_ in UV^35^	mmHg	42	
Hemoglobin Hb^41^	g/dL	12.2 ± 1.6	12.91 ± 1.38
Hematocrit Hct^41^	%	38.59 ± 3.94	40.88 ± 4.4
pH in UA^35^	-	7.29	
pH in UV^35^	-	7.35	
O_2_ transfer (OT)^c^	mL_O2_/L_blood_	55.82	59.04
O_2_ transfer rate (OTR)^d^	mL_O2_/min	3.72	6.52
CO_2_ transfer (CT)^c^	mL_CO2_/L_blood_	35.01	35.30
CO_2_ transfer rate (CTR)^d^	mL_CO2_/min	2.33	3.90
Max. pressure drop of art. placenta^e^	mmHg	20	

pO_2_: oxygen partial pressure, sO_2_: oxygen saturation, pCO_2_: carbon dioxide partial pressure, UA: umbilical artery, UV: umbilical vein.

^a^determined from a specified regression line in the respective reference

^b^derived from graph in ref. ^28^

^c^calculated using the equation by ref. ^42^ with values of refs. ^35,41^

^d^calculated by multiplying the respective transfer with the GA dependent UV flow in ref. ^22^

^e^conservative estimation (according to ref. ^43^ the natural placenta has a max. pressure drop of 30 mmHg)

#### Maximum extracorporeal blood volume

According to Kiserud et al.^28^, approximately 50% of the fetal blood volume resides in the placenta at mid-gestation, with the proportion declining with increasing GA from 26 weeks on. At 28 weeks of GA around 40% of fetal blood is still contained within the placenta. In the targeted treatment period, the overall blood volume nearly doubles from 55 mL to 96 mL.^29^ Hence, about 27.5 mL to 38.4 mL are physiologically used for the placental circulation at 24- and 28-weeks’ GA, respectively. However, these values apply to healthy fetuses, and EPI can suffer from pathologies like intrauterine growth retardation, which is associated with a reduced blood volume directed towards the placenta.^22^ We have therefore decided to restrict the priming volume of the artificial placenta to a maximum of 20 mL and to aim for at least partial priming with allogen fetal or adult blood.

#### Blood flow

The umbilical cord (UC) represents a natural interface and can serve as an access point to the central circulation.^20,30,31^ Thus, we consider the umbilical venous (UV) flow to determine the blood flow available for the extracorporeal circuit. In literature, considerably deviating values for the UV flow are reported—see Acharya et al.^32^ for an overview. For example, for 24 weeks of GA, UV flow mean values of 66.6 mL/min^22^ versus 118 mL/min^33^ have been published. One possible explanation for the variability is the dependence of the flow on the weight of the infant, which can highly vary even with the same GA. UV flow is therefore often normalized to fetal weight. Other factors such as differences in study design, ultrasound technique and equipment, measurement location, or operator expertise also have a strong influence on the quality of the measurements.^32^ Furthermore, it has to be taken into account that the UV flow (both absolute and normalized) is significantly lower in growth-restricted fetuses due to placental compromise as shown in Boito et al.^34^ and Kiserud et al.^22^ Under the premise of opting for a broadly applicable device, we have therefore specified blood flows from 50 mL/min to 150 mL/min for the oxygenator design.

#### Blood-gas values

As published by Motoyama et al.^35^, natural placental blood gas exchange increases the oxygen partial pressure pO_2_ from 19 mmHg in the umbilical arteries (UA) at 38% oxygen saturation (sO_2_) to 31 mmHg in the UV at 72% sO_2_. On the other hand, pCO_2_ is decreased from 51 mmHg (UA) to 42 mmHg (UV). Although large variations with up to two-digit standard deviations (see e.g., ref. ^36^) are also present here, the value ranges are supported by others^22,33,36–40^ and we decided on a source that provides as many values as possible in one. Nonetheless, the highest oxygen saturation in the UV reported in literature was 82% (for GA <31 week, mean value 61% with added standard deviation 21%^36^). In the perspective of a safe estimate and considering that the oxygen transfer can be easily reduced by adjusting the sweep gas flow, we have set the oxygen saturation threshold of our novel oxygenator to this value (82%). Based on the aforementioned blood gas values, the pH values in ref. ^35^ and the GA-specific hemoglobin and hematocrit values obtained by Forestier et al.^41^, we calculated the required oxygen and carbon dioxide transfers (OT and CT) using the respective equations provided by Khadka et al.^42^. The corresponding transfer rates, OTR and CTR, were then calculated by multiplying the results with the GA-dependent mean UV flows given in ref. ^22^. The resulting values are presented in Table 1.

#### Maximum pressure-loss

In Wang et al.^43^, blood pressure in the UA and UV is reported to be approximately 50 mmHg and 20 mmHg, respectively. Thus, the physiological pressure loss within the placental circulation amounts to 30 mmHg. However, this value may exceed the clinically recommended minimal mean arterial blood pressure of an EPI (which equals the GA in weeks according to a widely used rule of thumb), so we limited the maximum pressure loss in the extracorporeal circuit to less than 20 mmHg.

### Oxygenator design and manufacturing

**Fig. 1:**
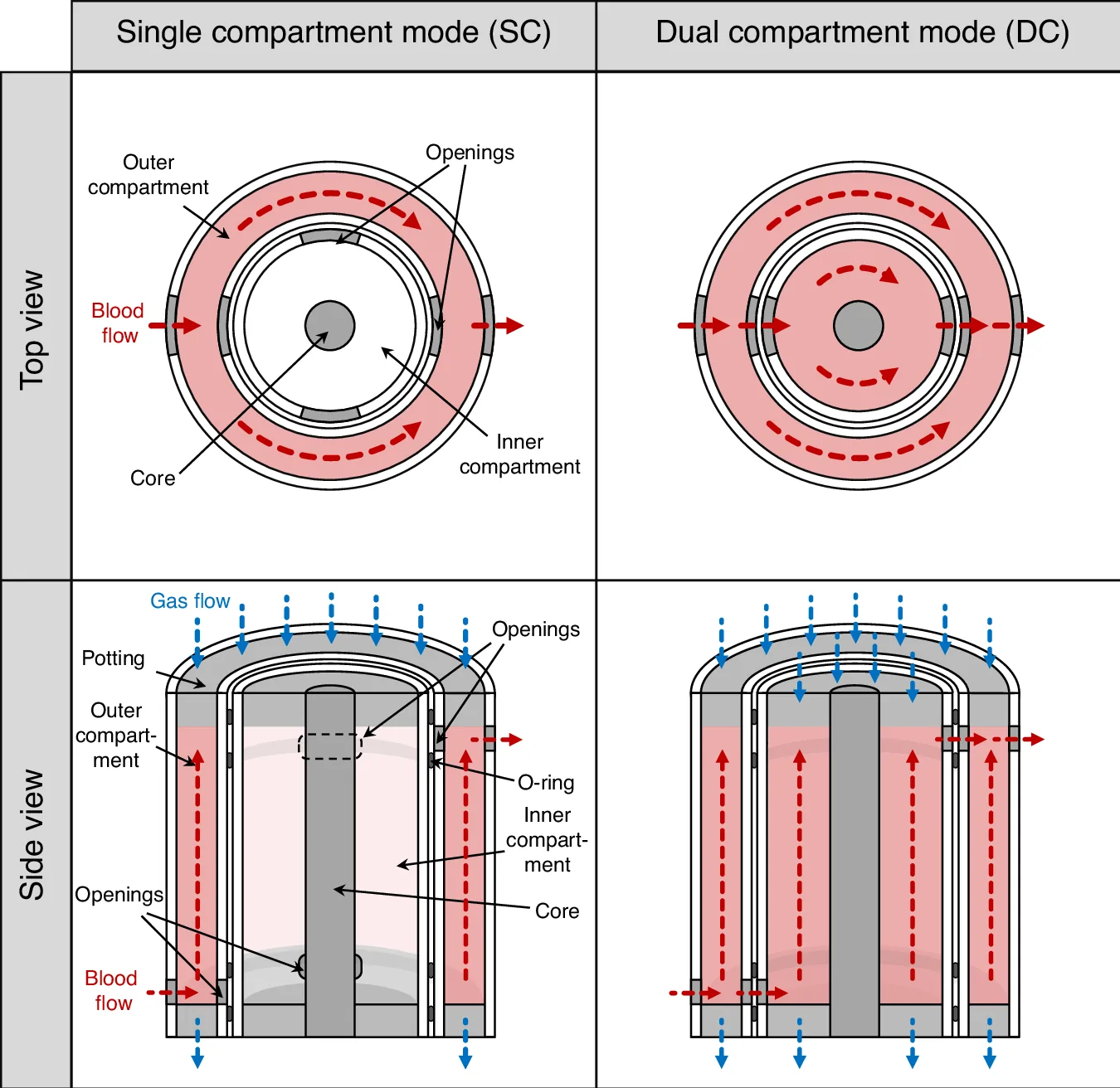
Functional sketch of the A-Maze-Ox. Left: top and side views of the oxygenator in single compartment mode (SC) - blood can only flow through the outer compartment; right: top and side view of the oxygenator in dual compartment mode (DC) - the inner compartment is rotated 90° such that the integrated openings overlap and the blood can flow through both compartments.

We chose a cylindrical design with wound hollow fibers inspired by the NeonatOx^15^ to avoid thrombus formation due to low flow rates in corner areas. The A-Maze-Ox consists of two concentric compartments, each of equal volume (5 mL), to prevent irregular flow due to pressure differences; see Fig. 1 for a functional sketch. In single compartment mode (SC), only the outer compartment is perfused. As the infant’s blood flow increases with growth, more gas transfer surface is required. To meet this demand, blood flow to the inner compartment can be opened during ongoing treatment by rotating the inner housing cylinder such that passages in the cylinder walls are lined up (dual compartment mode—DC). As these passages resemble a maze, we named our oxygenator accordingly: A-Maze-Ox. Two openings are located at the top and two at the bottom of the oxygenator (see Fig. 1 – side view). In the space between the two compartments, O-rings are inserted above and below each window.

An analytical determination of the gas exchange area necessary for adequate gas transfer requires empirical factors that are not yet available for our novel oxygenator design. Therefore, an approximation was carried out with commercially available small-volume oxygenators and the NeonatOx (priming volume range: 12–105 mL) considering their ratio of gas exchange area to dedicated maximum blood flow rate (see ref. ^27^ for an overview and further background). Taking into account that the physiological fetal pO_2_ demands are much lower than those after birth (Table 1) and considering our experience with the NeonatOx device, which already showed overly sufficient oxygen transfer, we derived a gas exchange area of 0.065 m² for each compartment.

The walls of the A-Maze-Ox are made of transparent polymethylmethacrylate (PMMA) to allow visual assessment of possible thrombus formation or shunt flow on the oxygenator wall. All components in this prototype, except the hollow fiber membranes, have been designed for multiple use to save cost and time. The single-layer hollow fiber membranes are composed of polymethylpentene (PMP; 3 M™ Membrana™, Wuppertal, Germany).

As there is no separate access to the inner compartment, it must be primed before use. Therefore, the oxygenator is first filled with saline solution in DC mode and then turned back to SC mode. The outer compartment can then be primed subsequently with blood to avoid blood dilution. Later, when adding the inner compartment to the blood flow, dilution inevitably takes place due to the design described. However, since the total blood volume of the infant is estimated to be 73 mL – 96 mL^29^, at the time more gas exchange area is required (26^th^ – 28^th^ week of GA), the dilution caused by 5 mL (volume of the inner compartment) saline solution would amount to only 5% – 7% and in the worst case, would result in a drop of hemoglobin concentration from 12.91 g/dL^41^ to 12.08 g/dL.

### Experimental setup

In terms of a proof-of-concept, the A-Maze-Ox was evaluated for its in-vitro gas transfer performance and pressure loss according to ISO 7199 (pCO_2_: 45 ± 5 mmHg, Hb: 12 ± 1 g/dL, sO_2_: 65 ± 5%, cGlu: 10 ± 5 mmol/L, and BE: 0 ± 5 mmol/L). We used abattoir porcine blood for which we had the approval of the competent veterinary authority in accordance with Article 23 of Regulation (EC) No 1069/2009. The blood was pretreated with 15.000 I.E Heparin, Refobacine (1.6 mL/L_blood_), 50% Glucose (1.8 mL/L_blood_) and 0.9% sodium chloride solution (6 mL/L_blood_). No human subjects were involved in the study.

**Fig. 2:**
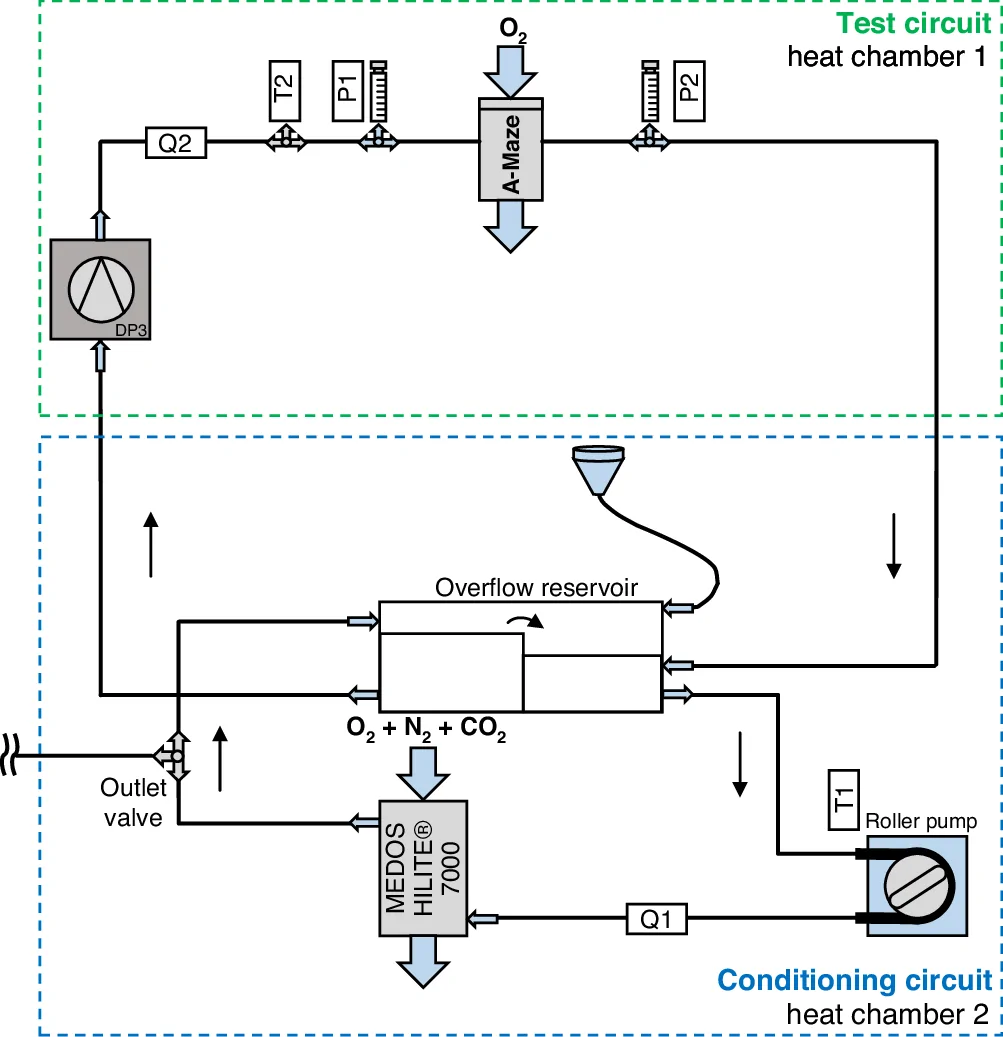
Schematic of the test setup. Top: test circuit in heat chamber 1 with the A-Maze-Ox driven by a DP3 rotary pump (MEDOS Medizintechnik AG, Heilbronn, Germany); bottom: conditioning circuit in heat chamber 2 for resetting blood inlet values with a MEDOS HILITE® 7000 oxygenator (MEDOS Medizintechnik AG, Heilbronn, Germany) driven by a roller pump; P1-2: pressure sensors, T1-2: temperature sensors, Q1-2: volume flow sensors.

**Fig. 3:**
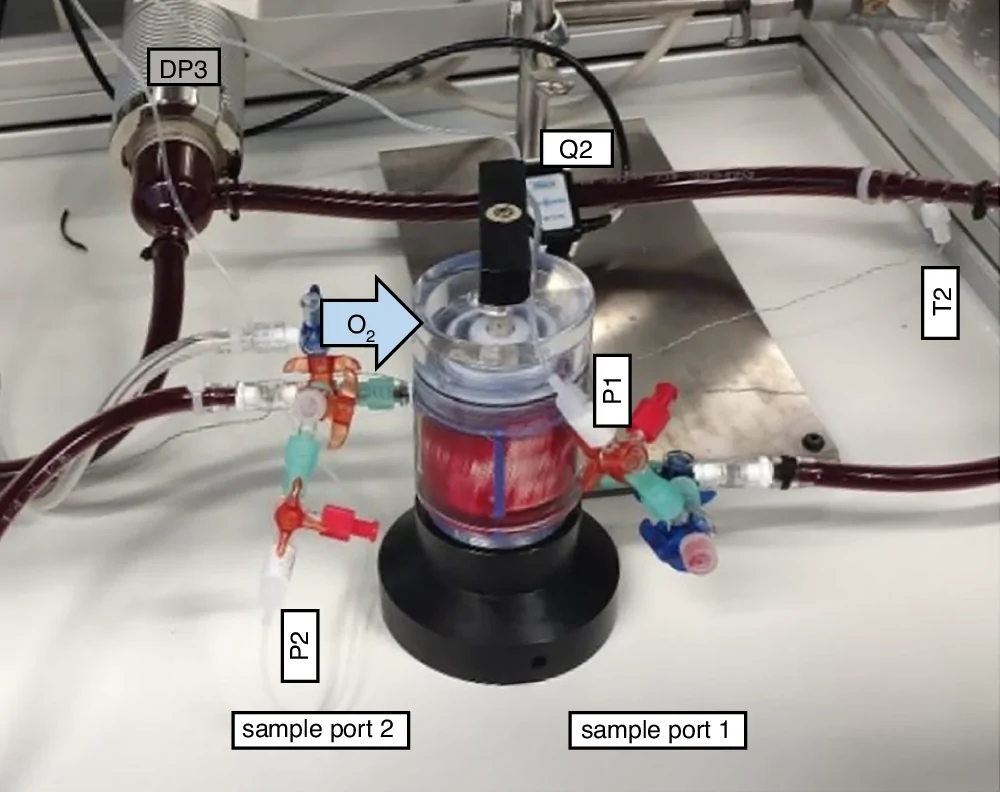
Photo of the test circuit with the A-Maze-Ox. The circuit is driven by a DP3 rotary pump (MEDOS Medizintechnik AG, Heilbronn, Germany); pressure sensors P1 at the oxygenator inlet and P2 at the oxygenator outlet, as well as the volume flow sensor Q2 and temperature sensor T2 for monitoring the circuit, are marked accordingly; oxygen (O_2_) is supplied via a connection in the cap of the oxygenator at a ratio of 2:1 relative to blood flow.

The experimental setup consists of a test- and a conditioning circuit connected by an overflow reservoir as shown in Fig. 2. Both circuits have separate heating chambers that ensure a blood temperature of 37 °C ± 1 °C in accordance with ISO 7199. In the test circuit (see Fig. 3), blood flows from the overflow reservoir through the A-Maze-Ox using a DP3 rotary pump (MEDOS Medizintechnik AG, Heilbronn, Germany). Oxygen is delivered through a connector at the cap of the oxygenator at a ratio of 2:1 relative to the blood flow. After oxygenation, the oxygenated blood returns to the reservoir and is processed through a conditioning circuit with another oxygenator (MEDOS HILITE® 7000, MEDOS Medizintechnik AG, Heilbronn, Germany) to reset the blood inlet values according to ISO 7199. Priming was performed with 0.9% sodium chloride solution. Different blood flow rates were considered for gas transfer performance evaluation to account for the infant’s increasing blood flow over time. As observed in a preliminary test, a drop in oxygen transfer performance is expected to occur at a blood flow rate of about 125 mL/min, so we decided to open the second compartment at this rate and test this operating point in both SC and DC mode. Thus, a total of five operating points were examined: 50 mL/min (SC), 100 mL/min (SC), 125 mL/min (SC), 125 mL/min (DC) and 150 mL/min (DC). For each operating point, 2 mL blood samples were taken at the sample ports before and after the oxygenator. Sampling is repeated after 6 min. This interval is determined by the time it takes for the blood to flow once through both circuits. To prevent adulteration, 1 mL was discarded before each sample. Blood samples were analyzed for sO_2_, pO_2_, and pCO_2_ using the ABL 825 FLEX blood gas analyzer (Radiometer GmbH, Krefeld, Germany). Measured values from both samples were averaged and gas transfer efficiency was calculated. Subsequently, with the oxygenator in DC mode, pressure was measured (Fig. 2: P1 and P2) at flows from 50 mL/min to 190 mL/min in 10 mL/min increments and the pressure difference was determined.

Data analysis was performed using MATLAB® (The MathWorks, Inc., Natick, MA), Version R2023a. Since only one test was performed to demonstrate the feasibility of the novel design, no statistical analysis was conducted.

## Results

**Fig. 4:**
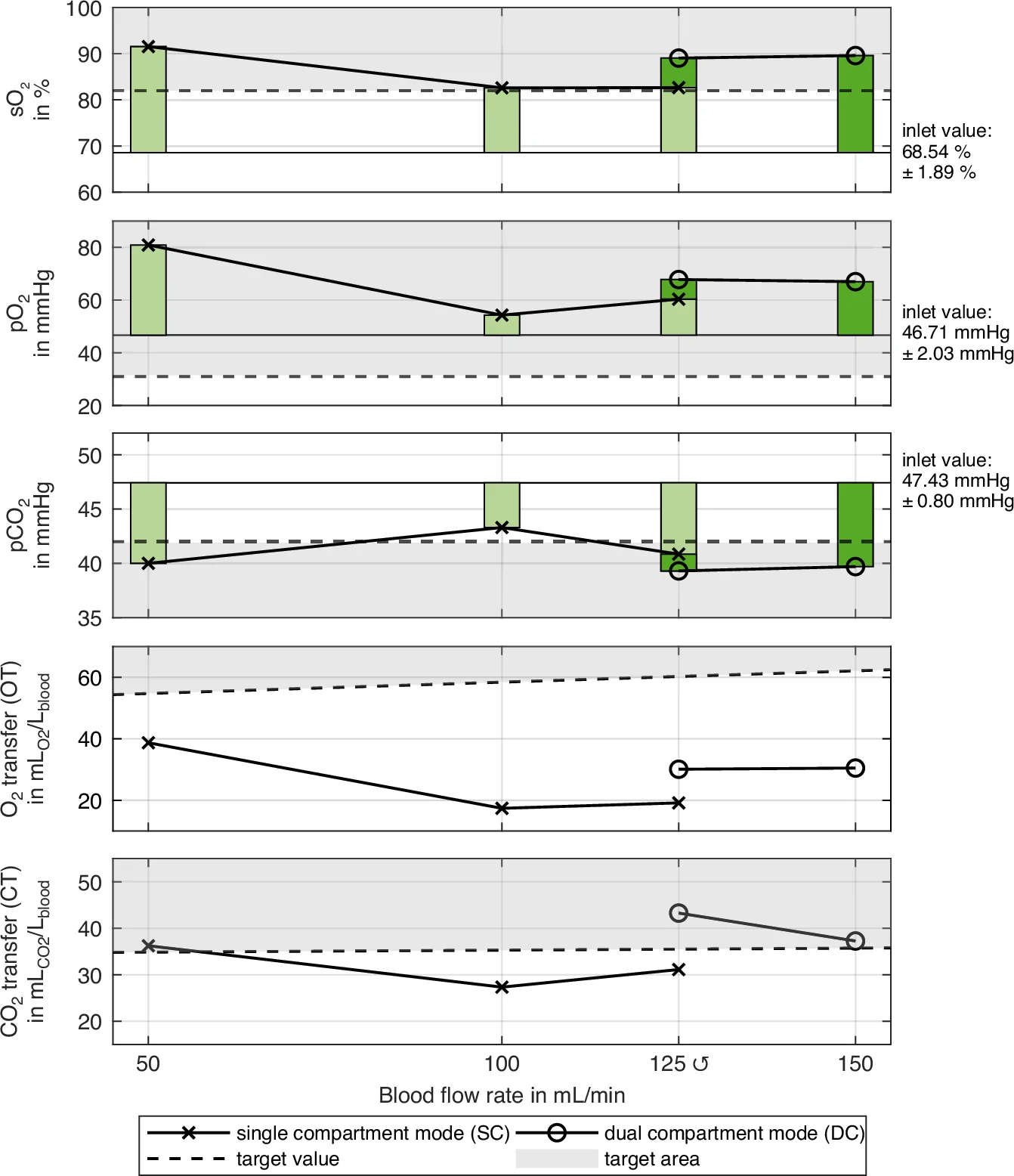
Blood gas values sO_2_, pO_2_ and pCO_2_ measured behind the oxygenator, as well as O_2_ and CO_2_ transfer plotted over the blood flow rate. Inlet values for the respective blood gas measurements were averaged over all samples (*n* = 10) and given as mean baselines; target values are indicated by dashed lines—if target values are given for specific GAs, linear interpolation was used to define a target line; areas above or below (in the case of pCO_2_) which show the range in which our oxygenator performs well, are highlighted in gray (target area); values for the oxygenator in single compartment (SC) and dual compartment mode (DC) are shown as x and o, respectively.

Blood gas values and Oxygen transfer performance In Fig. 4, the blood gas values sO_2_, pO_2_ and pCO_2_ measured behind the oxygenator as well as the oxygen (OT) and carbon dioxide transfer (CT) are depicted. Please note: The blood inlet values for sO_2_ as specified by ISO 7199 were minimally exceeded twice (71.7% ( + 1.7%) at a blood flow of 100 mL/min and 70.1% ( + 0.1%) at 125 mL/min – both only one sample and in SC mode). These values were nevertheless included in the evaluation, as no excessive distortion of the results was to be expected. In the figure, the respective target values are indicated by dashed lines and the areas above or below (in case of pCO_2_), which show the range in which our oxygenator performs well, are highlighted in gray.

Figure 4—Blood gas values sO_2_, pO_2_ and pCO_2_ measured behind the oxygenator as well as O_2_ and CO_2_ transfer plotted over the blood flow Q. Inlet values for the respective blood gas values were averaged over all samples (*n* = 10) and given as mean baselines; target values are indicated by dashed lines—if target values are given for specific GAs, linear interpolation was used to define a target line; areas above or below (in case of pCO_2_) which show the range in which our oxygenator performs well, are highlighted in gray (target area).

Starting with 91.5% saturation at a blood flow rate of 50 mL/min, sO_2_ dropped to 82.6% at 100 mL/min and 82.7% at 125 mL/min. By opening the second compartment, we were able to increase sO_2_ to almost 90% again, consistently exceeding the target value of 82%. Regarding pO_2_, an increase of approximately 12% from 60.35 mmHg to 67.80 mmHg was observed when adding the second compartment at 125 mL/min. Before, a slight drop in pO_2_ could be noticed at 100 mL/min. The arterial target value of 31 mmHg was achieved across all blood flow rates. However, it should be noted that the mean inlet value recorded in our test performed according to ISO 7199 (indicated by a mean baseline of 46.71 mmHg ± 2.12 mmHg in Fig. 4) was already above this target value and far higher than the initial value of 19 mmHg in the UA of the fetus. The pCO_2_ could be decreased by 3.8% from 40.85 mmHg to 39.3 mmHg upon switching from SC to DC mode at 125 mL/min. At 150 mL/min, pCO_2_ slightly increased again to 39.7 mmHg. Except for one small outlier at 100 mL/min with a value of 43.4 mmHg, the target range of <42 mmHg was always met throughout the measurements. It is necessary to emphasize here that the inlet value of 47.43 mmHg ± 0.80 mmHg is already considerably lower than the initial value in the fetal UA of 51 mmHg but still above the target value.

The O_2_ transfer (OT) of the A-Maze-Ox in SC mode started with 39.095 mL_O2_/L_blood_ and then considerably dropped to 17.44 mL_O2_/L_blood_ and reached 19.30 mL_O2_/L_blood_ at 125 mL/min. Addition of the second compartment (DC mode) increased the OT performance by 57.06% to 30.32 mL_O2_/L_blood_ with a maximum of 30.69 mL_O2_/L_blood_ at 150 mL/min. However, the target values of 55.96 mL_O2_/L_blood_ and 59.19 mL_O2_/L_blood_ for an infant at 24^th^ and 28^th^ week of GA, respectively, could not be achieved in our measurements. In contrast, the target values for CO_2_ transfer (CT), which amount to 35.01 mL_CO2_/L_blood_ (24^th^ weeks of GA) and 35.30 mL_CO2_/L_blood_ (28^th^ week of GA) were exceeded in SC mode at 50 mL/min. Although CT subsequently fell below the target line for higher flow rates in SC mode, it increased by 35.59% with the addition of the second compartment, again surpassing the target.

**Fig. 5:**
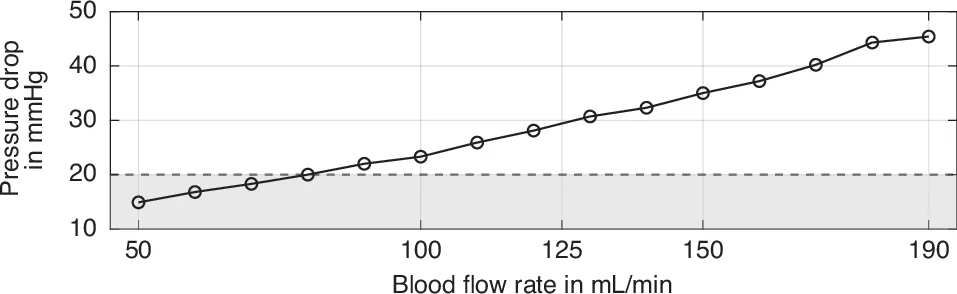
Pressure drop in DC mode plotted over the blood flow rate. Targeted maximum value of 20 mmHg indicated by the dashed line; gray area marks the target range.

Pressure loss The pressure loss (Fig. 5) was measured in DC mode to 14.9 mmHg at 50 mL/min blood flow and increased with increasing flow rate. The target criterion of 20 mmHg was already exceeded at 90 mL/min, culminating in 45.4 mmHg pressure loss at 190 mmHg.

## Discussion

We designed and tested a novel small-volume oxygenator prototype that allows to adjust the gas exchange area without changing the device. The maze-inspired design with concentrically arranged compartments enables the volume to be increased from 5 mL to 10 mL and the gas exchange area from 0.065 m² to 0.13 m². Although the significance of the in-vitro test results is limited by the small number of samples, the test provides insights into the functionality of the new oxygenator concept.

The feasibility of adjusting the gas exchange area has been demonstrated, with oxygen (OT) and carbon dioxide transfer (CT) performances increasing by 57.06% and 35.59%, respectively, when adding the second compartment. However, these values are lower than expected; for instance, we had assumed an increase in OT capacity of approximately 100%. A potential explanation for this discrepancy might be an undetected shunt flow occurring on an internal wall that is not visible during testing. Furthermore, the target values for OT (55.96 mL_O2_/L_blood_ at 24^th^ week of GA; 59.19 mL_O2_/L_blood_ at 28^th^ week of GA) were not achieved, with a maximum OT of only 39.095 mL_O2_/L_blood_ (SC mode, 50 mL/min). In contrast, CT results were promising as the target (35.01 mL_CO2_/L_blood_ – 35.30 mL_CO2_/L_blood_) was surpassed in SC mode at a blood flow rate of 50 mL/min and for all flow rates in DC mode. The target values for sO_2_ and pO_2_ were considerably exceeded in both modes, while pCO_2_ showed one small outlier at a blood flow rate of 100 mL/min in SC mode. However, since the target value was clearly reached at 50 mL/min and 125 mL/min and a similar outlier was recorded for pO_2_ at this operating point, it is reasonable to assume that this value is due to a measurement error, an evaluation error in the ABL device or an undetected shunt flow.

It is important to note that the inlet values for sO_2_ = 65% ± 5% and pCO_2_ = 45 mmHg ± 5 mmHg, required by ISO 7199 and applied in our test, do not align with the initial fetal values in the UA, which are in the order of 38% and 51 mmHg, respectively.^35^ Consequently, our resulting inlet value of pO_2_ (46.71 mmHg ± 2.12 mmHg) was already above the desired target value of 31 mmHg in the UV.

Further aspects must be taken into account regarding the physiologically determined requirements: The in-vitro tests were conducted with (adult) porcine blood. The hemoglobine of this blood has a lower oxygen affinity than fetal blood. This results in a rightward shift of the oxygen binding curve and a reduction in achievable sO_2_ at a given pO_2_.^35,44^ The sO_2_ measured here in compliance with ISO 7199 is therefore probably underestimated compared to an application in the human neonate. Additionally, the above-mentioned use of the blood inlet conditions according to ISO 7199 with a much higher initial pO_2_ than that of a fetus results in a reduced partial pressure gradient across the oxygenator and thus a reduced OT performance. Given all these constraints, it is justified to assume that the OT performance achieved with the A-Maze-Ox might be considerably higher in a clinical application. The same holds true for CT. Nevertheless, the design should be revised with a focus on improved OT and CT. For future testing, it is also advisable to consider using fetal parameters instead of the inlet values specified in ISO 7199. This approach would account for pressure gradients and provide a more accurate assessment of the performance in the context of EPI.

Compared to our previously published oxygenator,^26^ we achieved a considerably better CO_2_ elimination, but unfortunately, the pressure loss over the oxygenator was much higher than expected and already exceeded the maximum value of 20 mmHg at a blood flow of 90 mL/min. This also needs to be investigated and considered when revising the design, particularly given that cannulation and tubing will cause additional pressure loss.

For medical applications, the oxygenator ought to be operable for the entire treatment period which is currently considered to be at least four weeks. Any change of the device would cause hemodilution and transient hypoxemia. This long-term application in a patient with unique growth dynamics entails two main requirements: variable gas exchange surface and sufficient long-term function. We have shown that it is possible to design an oxygenator that “grows” over time to meet the increasing oxygen demand, but we still need to work on enabling long-term use in terms of induced hemolysis and the need for anticoagulation. Since cannulation, for example, also has a significant impact on these aspects, the A-Maze-Ox must always be considered holistically in the context of the overall artificial placenta system.

## Conclusion

We here propose the A-Maze-Ox – a novel small-volume dual-compartment oxygenator that allows the gas exchange area to be increased without changing the device. We tested the feasibility in terms of gas transfer and pressure drop in a pilot study. The pressure drop was too high at higher flow rates. Switching from single to dual compartment mode increased oxygen transfer by 57.06% and carbon dioxide transfer by 35.59%. The absolute oxygen transfer performance was not sufficient yet, but our design showed improvement of CO_2_ elimination compared to our previous prototype.^26^ Based on these results, we are currently working on revising the design to improve performance and pressure loss and conducting more extensive blood gas transfer and hemolysis testing to ensure functionality and safety of the device. Depending on the upcoming findings, further design iterations may be necessary (possibly accompanied by simulations), again followed by in-vitro testing in accordance with ISO 7199. In addition, before translation into clinical practice, long-term tests based on the actual intended duration of use and, finally, in-vivo animal studies must be carried out. Although several steps still need to be taken, the present study already shows the potential for a growing oxygenator in future artificial placenta applications.

## Data Availability

The data sets generated during and/or analyzed during the current study are available from the corresponding author on request.
